# Health Behaviours As a Mechanism in the Prospective Relation between Workplace Reciprocity and Absenteeism: A Bridge too Far ?

**DOI:** 10.1371/journal.pone.0141608

**Published:** 2015-11-02

**Authors:** Bart De Clercq, Els Clays, Heidi Janssens, Dirk De Bacquer, Annalisa Casini, France Kittel, Lutgart Braeckman

**Affiliations:** 1 Department of Public Health, Ghent University, Ghent, Belgium; 2 Psychological Sciences Research Institute, Université catholique de Louvain, Louvain-la-Neuve, Belgium; Medical University Vienna, AUSTRIA

## Abstract

**Background:**

The persistent lack of evidence on causal mechanisms between social capital and health threatens the credibility of the social capital—health association. The present study aims to address this ongoing problem by investigating whether health behaviours (i.e. smoking, alcohol consumption, and physical activity) mediate the prospective relation between workplace reciprocity and future sickness absence.

**Methods:**

A cohort of 24,402 Belgian employees was followed up during 12 months for sickness absence. Workplace reciprocity was measured with four indicators—colleague help, colleague interest, supervisor help, and supervisor concern. Three types of multilevel mediation models were applied.

**Results:**

Overall, workplace reciprocity negatively related to high sickness absence (≥ 10 days) mainly independently from health behaviours. Uniquely, colleague interest positively related to smoking (OR = 1.058, 95% CI = 1.019, 1.098) and smoking in turn, positively related to sickness absence (OR = 1.074, 95% CI = 1.047, 1.101). No behavioural pathways could be identified between company-level reciprocity and sickness absence, and company-level health-related behaviours did not mediate the relation between company-level reciprocity and individual sickness absence.

**Conclusions:**

These results suggest that both social capital and health behaviours are relevant for employee health, but health behaviours seem not to be the underlying explanatory mechanism between workplace reciprocity and health.

## Introduction

It is widely recognized that beyond the individual, social environments such as neighbourhoods and workplaces may promote or constrain the practice of healthy lifestyles that ultimately lead to health and illness [1]. For the past two decades, epidemiologists and public health researchers discussed the theoretical and empirical value of social capital as such a social-environmental factor [2–4]. Despite a significant disciplinary advance, the social capital—health association is characterized by two ongoing problems: data accessibility bias and absence of causal mechanisms.

The elastic definition of social capital [5] tolerated a large diversity of measurement instruments [6], which may have generated findings that are biased by the accessibility of data [7]. Secondary data-analysis is common practice in scientific research, but as a result, researchers tend to use data obtained for other purposes rather than using variables designed for measuring social capital [8]. This limitation could feed the impression that some dimensions (e.g. trust and social participation) are more related to health outcomes than other less popular indicators such as reciprocity. Although it is considered as a key part of the concept of social capital [9–11], reciprocity remains misunderstood, undertheorized, and rarely measured [12]. Traditionally, social capital has been studied in neighbourhoods, societies, and even nations [13], however recently the workplace has become an important social context [14]. In line with the classical measure of Kawachi et al. [15], previous research outlined a theoretical model for assessing reciprocity in the workplace [16]. The model integrates three common classifications from both social capital and social support theory. A first distinction in the conceptualization and measurement of social capital is drawn between “horizontal” and “vertical” components [17]. Horizontal reciprocity reflects ties that exist among individuals or groups of equals or near-equals, and vertical (linking) reciprocity refers to interactions across explicit, formal or institutionalized power or authority gradients in society such as relationships between employees and supervisors [18]. Orthogonal to the distinction between horizontal and vertical components, reciprocity can be decomposed into “emotional” and “instrumental” components. Emotional reciprocity involves the provision of empathy, trust and caring, whereas instrumental reciprocity refers to practical help [19]. A third distinction concerns the level of analysis—whether reciprocity is treated as an individual-level attribute or as a workplace-level characteristic [2]. In sum, the combined associations of both the horizontal-vertical and emotional-instrumental components can be simultaneously investigated both on worker (level 1) and on workplace level (level 2), resulting in a multilevel statistical model.

Prospective multilevel studies investigating the effect of social capital on health are limited [20] and have barely tested causal mechanisms or underlying pathways in which social capital may affect health [7]. Most studies examined association and then referred to hypothetical pathways or mechanisms derived from ecological studies or reviews that do not actually provide evidence of any mechanism. Of the few studies that were conducted in the context of workplaces [21–27], one study showed that the association between workplace social capital and hypertension was partially mediated by obesity and alcohol consumption in men but not in women [25]. Other studies have also used health behaviours as the mediating factor to explain the relation between individual [28] and contextual [29–31] social capital and health but this is merely cross-sectional evidence from neighbourhood settings. Note that these studies presume directionality from social capital to health through health behaviours, however a critical point is that most of the associations between social capital, health behaviors, and health could also be reversed. For example, shared interests—expressed in health behaviour such as physical activity—could be an important determinant of social capital. In this context, Choi et al. [7] listed five points that future studies should address: (i) adjust for area-level confounders instead of only basic characteristics of the individual, (ii) incorporate potential pathway variables as mediators instead of simply adjusting for them, (iii) undertake proper mediation analysis, (iv) apply multilevel modeling to capture social capital’s multidimensional nature, and (v) model time-varying confounders instead of only baseline variables.

In line with Choi et al. [7] the present study aims to address both issues outlined above (i.e. data accessibility bias and absence of causal mechanisms) with an empirical test of the health behaviour pathway [32] as a mechanism in the prospective relation between workplace reciprocity at baseline (t) and sickness absence at follow up (t+1). Although sickness absence is a complex multifactorial phenomenon, several studies have demonstrated a clear association between ill-health and sickness absence [33]. Furthermore, there is also evidence that objective sickness absence data predict mortality rates as well as other established indicators of health [34]. Therefore, sickness absence records can be used as a global measure of health and functioning among working populations [33,34].

**Fig 1:**
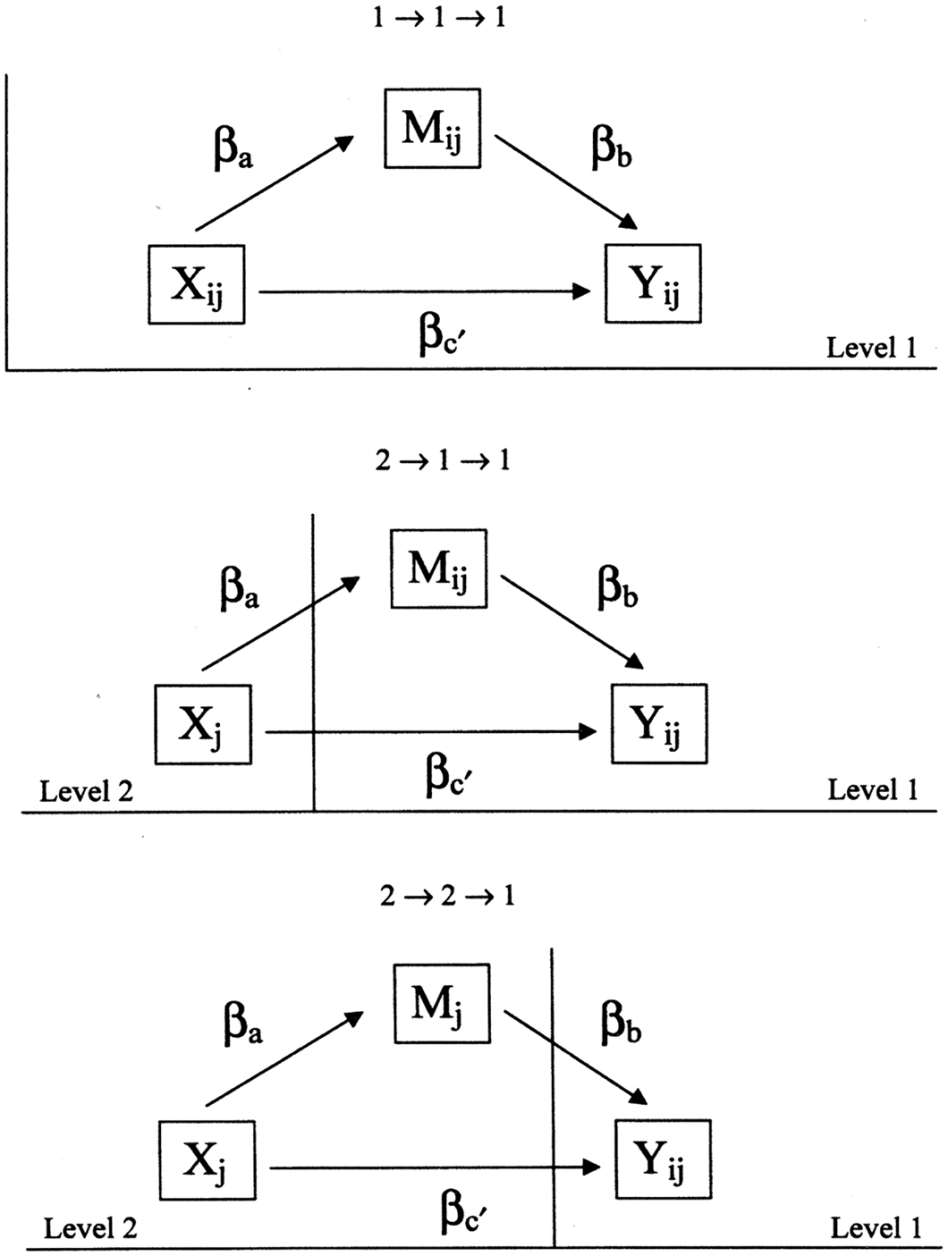
Three types of mediational models adopted from Krull & MacKinnen, 2001.

Following Krull and MacKinnon [35], we formulated three mediation hypotheses (Fig 1). The first mediation hypothesis is labeled 1→1→1, indicating that reciprocity (*X*_*ij*_), health behaviour (*M*_*ij*_), and sickness absence (*Y*_*ij*_) variables are conceptualized at the first or individual level of the data. For example, this type of mediation conceptualizes smoking uniquely as individual risk behaviour and tests the individual dimension of the workplace reciprocity model [16]. The second hypothesis (2→1→1) assumes that reciprocity at the company-level determines sickness absence trough individual health behaviour. The final hypothesis (2→2→1) conceptualizes both health behaviour and reciprocity as group characteristics so that the norms of reciprocity in the company influence individual sickness absence through the prevalence of certain health behaviours within the company.

## Materials and Methods

### Study population

BELSTRESS is a large epidemiological cohort study on job stress, cardiovascular risk factors and sickness absence [36,37]. Baseline data (t) in the present study result from the merger of BELSTRESS I and BELSTRESS III study. BELSTRESS I was conducted between 1995 and 1998 (n = 21,419) corresponding with 87.8% of the pooled database) [36]. A participation rate of 48% was reached. BELSTRESS III was conducted in 2004 (n = 2,983 corresponding to 12.2% of the pooled database) [37]. The response rate was 30.4%. The pooled sample comprises information on 24,402 workers (6,701 women) from 32 companies or public administrations across Belgium. Follow-up data (t+1) on sickness absence were gathered for both BELSTRESS I and BELSTRESS III after 12 months. Within the participating companies, all workers aged 30 to 59 years were invited to volunteer in the study. Although the sample was not totally representative for the Belgian workforce, the study cohort covers a broad range of companies and occupational groups. Data were collected by means of standardized questionnaires containing sociodemographics, work-related, and lifestyle factors, and health perception. All participants gave their written informed consent before inclusion in the project. BELSTRESS was approved by the ethics committees of Ghent University Hospital and the Faculty of Medicine of the Free University of Brussels.

### Reciprocity variables

The measurement of reciprocity as a cognitive aspect of social capital was based on the classical measure of Kawachi et al. [15] but additionally differentiates between several forms of reciprocity. Four indicators—colleague help, colleague interest, supervisor help, and supervisor concern—from the Job Content Questionnaire [38] were used to measure all dimensions of the reciprocity at work model [16]. Colleague help was measured by the following statement “My fellow workers are helpful in getting the job done” (horizontal instrumental reciprocity), colleague interest “People I work with take a personal interest in me” (horizontal emotional reciprocity), supervisor help “My supervisor is helpful in getting the job done” (vertical instrumental reciprocity), and supervisor concern “My supervisor is concerned about the welfare of those under him” (vertical emotional reciprocity). All items were scored on a four-point rating scale (1 = strongly disagree, 4 = strongly agree). Four P versus P plots that plot the cumulative probability of each indicator against the cumulative probability of a normal distribution were fitted to inspect the distribution of our indicators. Data points all fell very close to the “ideal” diagonal line, indicating that these indicators were normally distributed. Since these indicators were originally measured through individual responses, they automatically represent the individual dimension of reciprocity at work. The contextual dimension of reciprocity, on the other hand, was measured based on aggregated individual answers for every indicator (company level mean scores).

### Behavioural mediators

We analyzed three different health behaviours: smoking, alcohol consumption and leisure time physical activity [39]. Smokers were defined as those who regularly smoke cigarettes, cigars or pipes. Excessive alcohol consumption was defined as an average of more than three alcohol units per day for men and more than two units per day for women [40]. Leisure time physical activity was assessed on a four-point rating scale (1 = no weekly activity, 2 = only light physical activity during most weeks, 3 = heavy physical activity during 20 min or more once or twice per week, 4 = heavy physical activity during 20 min or more three times or more per week). Smoking and excessive alcohol consumption were defined as binary variables in the analysis, and leisure time physical activity was included as continuous variable.

### Follow-up of sickness absence

In collaboration with the personnel administration departments of the participating companies, information on absenteeism was gathered during a follow-up period of 12 months. Because the recording of sickness absence in Belgium is strictly ruled, requiring medical certification, we may assume that the sickness absence registration was highly accurate. Since former research clearly demonstrated a relationship between social capital and health [23,25] and depression [16,22,27], we hypothesized that workplace reciprocity possibly harms the health of the worker, rather than it would solely reflect coping behavior as an attempt to escape from a negative work environment. Therefore, we selected a measure which includes the long-term sickness absence, which reflects the health status of workers [33,34]. Persons who were absent for at least 10 days during the registered period (i.e. the upper quartile of the distribution of the total annual sickness days) were classified as having high sickness absence [41].

### Background characteristics

The data source was indicated by a binary variable (0 = BELSTRESS I; 1 = BELSTRESS III). Age was categorized into three groups (30 to 39; 40 to 49; and 50 to 59), with persons aged 30 to 39 years as the reference category. Educational level was assessed by the highest level of completed education based on a 6-point rating scale (1 = primary school, 2 = first half of secondary school, 3 = secondary school, 4 = extra year of specialization in secondary school, 5 = higher education, and 6 = university). Higher education refers to both vocational courses (only bachelor) and academic courses (bachelor and master). University is more specific and refers to the academic master degree. Occupation was assessed according to the International Standard Classification of Occupations code [42] and classified in three categories: 1 = executives, 2 = white collar, and 3 = blue collar (reference: executives). Health perception was measured with the current health index [43] which is a score computed from the VOEG scale (Vragenlijst Over Ervaren Gezondheid) [questionnaire on health perception], a Dutch scale built up from 13 closed questions each having two outcomes (0 = no, 1 = yes) and thus adding up to a scale between 0 and 13 [44]. Example items are: “Do you often suffer from headaches?; Do you often suffer from back pain?; Do you often feel tired? Do you occasionally suffer from pain in the chest or stomach area?”.

### Statistical analysis

To examine the several behavioural pathways between reciprocity defined both at employee- and workplace level and future high sickness absence, three types of mediation models were estimated in two steps. Mplus 7.11 [45] was used to implement the models.

#### Step 1: single-level path model (1→1→1)

Recursive path analysis was used as it enables dealing with complex social variables and their interrelations. The starting point for our model trimming approach was a complex just-identified model that tests the theory that health behaviours (smoking, drinking and physical activity) explain the prospective relation between individual-level reciprocity and high sickness absence. Therefore, the model included direct and indirect paths from all individual-level reciprocity variables to the sickness absence outcome via the different health behaviour variables. Bootstrapping was used to compute bootstrap standard errors for the indirect effects [46]. The model also included correlations and correlated error terms between all reciprocity variables. Next, the model was simplified by eliminating paths. All models were adjusted for the data source, gender, age, education, occupational status and health perception by regressing the dependent variable and the mediators on these covariates. In contrast to the Krull and MacKinnon typology [35], our baseline individual-level mediation model was not estimated within a multilevel framework for computational reasons (i.e. the more efficient single level weighted least squares means and variance adjusted (WLSMV) estimation enables more relevant model fit information and modification indices). Consequently, the 1→1→1 model could better be described as (*X*_*i*_)→(*M*_*i*_)→(*Y*_*i*_). Three fit indices were used to evaluate the models: (1) the chi-square value of model fit; (2) the Root Mean Square Error of Approximation (RMSEA) of the model; and (3) the Comparative Fit Index (CFI) [47]. Values below .05 on the RMSEA and a value of .90 or greater on the CFI were considered as indicative of a good fit. We modeled the most constrained model that has the relative best fit indicated by its chi-square value. To avoid fully data driven path models, we restricted our analyses to model modifications supported by the literature. Model coefficient estimates were converted into odds ratios (ORs) with 95% confidence intervals (CIs).

#### Step 2: multilevel path models (2→1→1 & 2→2→1)

The implementation of mediational pathways is not straightforward within the conventional approach to multilevel modeling [48]. Building on Muthén and Asparouhov’s [49] multilevel structural equation modeling (MSEM) mathematical framework, Preacher et al. [50] developed a MSEM framework for testing multilevel mediation. MSEM allows to disentangle within- and between-group effects and test the significance of indirect effects occurring at the contextual level. The developed models could best be described as multilevel path models since we did not create latent factors. A numerical integration algorithm was used to fit two-level random intercept models via maximum likelihood estimation with robust standard errors (MLR). A model building approach was applied here with increasing complexity by taking contextual-level workplace reciprocity into account. Models were evaluated on the basis of their efficiency using Akaike’s Information Criterion (AIC) and the more conservative Bayesian information criterion (BIC) [51]. Lower AIC and BIC values indicate better fit to the data.

## Results

### Demographics

Table 1 depicts the individual demographic information of the sample. The study sample contains 24,402 employees nested within 32 companies with an average cluster size of 677 individuals per company. A total of 6391 cases (26%) with high sickness absence duration were identified. Women showed more sickness absence than men (χ^2^ = 194.70, df = 1, p < 0.001).

**Table 1:** Descriptive statistics of the participants by dependent follow up (t+1) and independent baseline (t) variables (N = 24402).

	All	Women	Men	*p-value* *
	*N* = 24402	*N* = 6701	*N* = 17701	
*Independent baseline (t) variables*				
Study (%)				< 0.001
BELSTRESS I	88	76	92	
BELSTRESS III	12	24	8	
Age (mean (SD), range 30–59)	45 (6.08)	44 (5.96)	46 (6.07)	< 0.001
Educational level (%)				< 0.001
Primary school	40	34	42	
Secondary school	31	37	29	
Higher education	29	29	29	
Occupation (%)				< 0.001
Blue collar	32	18	37	
White collar	51	70	43	
Executives	17	12	20	
Current health index (mean (SD), range 0–13)	4.68 (3.35)	5.87 (3.47)	4.23 (3.20)	< 0.001
Reciprocity (mean (SD), range 1–4)				
Colleague interest	2.94 (0.58)	2.94 (0.60)	2.94 (0.57)	n.s.
Colleague help	2.99 (0.58)	2.95 (0.61)	3.00 (0.56)	< 0.001
Supervisor concern	2.70 (0.79)	2.71 (0.79)	2.69 (0.79)	n.s.
Supervisor help	2.65 (0.75)	2.64 (0.75)	2.65 (0.75)	n.s.
Health-related behaviours				
Current smoking (%)	28	27	28	< 0.05
Excessive alcohol consumption (%)	21	15	23	< 0.001
Leisure time physical activity (mean (SD), range 1–4)	2.23 (0.93)	1.92 (0.87)	2.34 (0.94)	< 0.001
*Dependent follow-up (t+1) variable*				
High sickness absence duration (%)	26	33	24	< 0.001

* result from chi² or t-test,

n.s. = not significant

#### Step 1: single-level path model (1→1→1)

**Fig 2:**
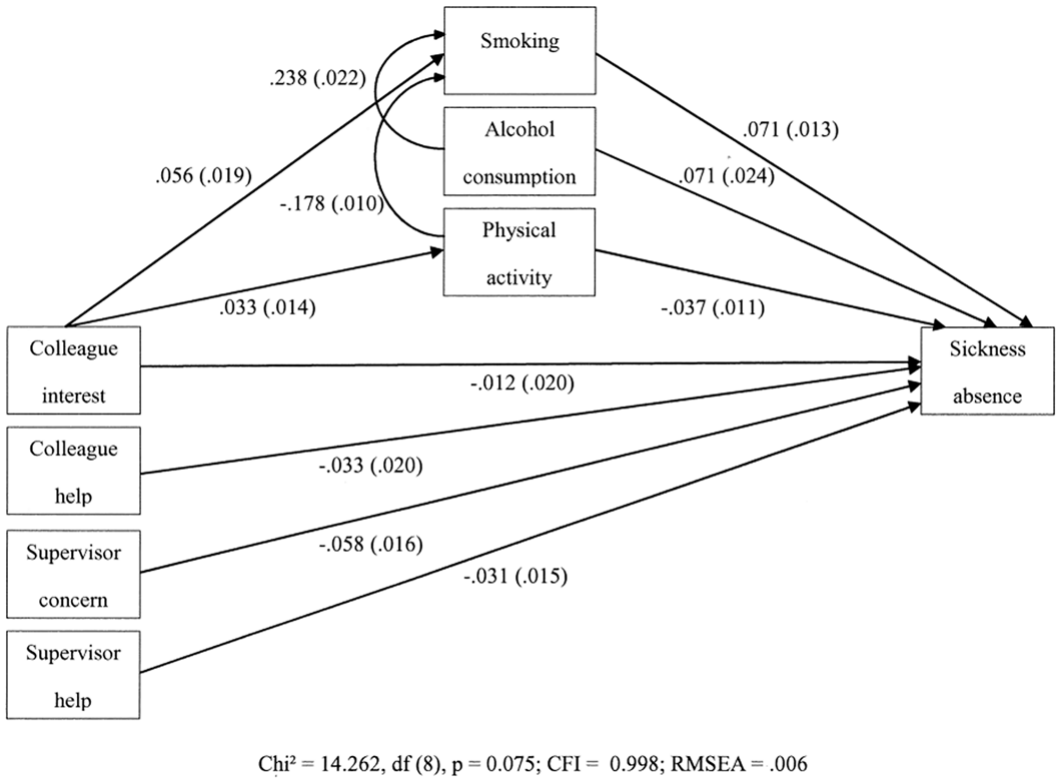
Single-level multiple mediation model (1→1→1).

The theoretical single-level model with four individual-level reciprocity variables and three mediating health behaviours was estimated following a model trimming approach. Fig 2 presents a simplified version of the core model without control variables and their covariance structure. Within the criteria, the final single-level model showed good fit to the data (χ^2^ = 14.262, df (8), p = 0.075; CFI = 0.998; RMSEA = .006). Only reciprocity variables that refer to the supervisor were significantly and negatively associated with high sickness absence. Supervisor concern (OR = 0.944, 95% CI = 0.915, 0.974) was the strongest protective factor for sickness absence: for one unit of increase on supervisor concern the odds of sickness absence decreases with 5.6%. The decrease in the odds of sickness absence for supervisor help (OR = 0.969, 95% CI = 0.941, 0.998) was 3.1%. Nevertheless, only colleague interest indirectly related to high sickness absence via smoking behaviour: colleague interest positively related to smoking (OR = 1.058, 95% CI = 1.019, 1.098) and smoking in turn, positively related to high sickness absence (OR = 1.074, 95% CI = 1.047, 1.101). The specific indirect effect of the behavioural smoking pathway was statistically significant at the 0.05 level (Table 2). Colleague interest also positively related to physical activity (OR = 1.034, 95% CI = 1.006, 1.062) and physical activity in turn, negatively related to high sickness absence (OR = 0.964, 95% CI = 0.943, 0.985). However, the specific indirect effect of the physical activity pathway was not significant. Excessive alcohol consumption directly related to high sickness absence (OR = 1.074, 95% CI = 1.024, 1.125). Significant interrelations between different health behaviours were also found: alcohol consumption was positively associated with smoking (OR = 1.269, 95% CI = 1.215, 1.325) and physical activity was negatively associated with smoking (OR = 0.837, 95% CI = 0.821, 0.854).

**Table 2:** Specific indirect effects for the multiple mediation models.

Effect	b	S.E.
*Single-level path model (1→1→1)*		
Baseline (t) colleague interest → baseline (t) smoking → follow-up (t+1) sickness absence	0.004	0.002*
Baseline (t) colleague interest → baseline (t) physical activity → follow-up (t+1) sickness absence	- 0.001	0.000^n.s.^
*Multilevel path model (2→1→1)*		
-	-	-
*Multilevel path model (2→2→1)*		
-	-	-

* *P* < 0.05

^n.s.^not significant

#### Step 2: multilevel path models (2→1→1 & 2→2→1)

The final single-level path model was used as a baseline model with which more complex multilevel path models were compared. The model with the lowest AIC and BIC values was interpreted. The variance partition coefficient revealed that 4.7% of the differences in high sickness absence were attributable to differences between companies. No contextual reciprocity effects were found and the between-company variance could also not be explained by compositional differences in reciprocity. Consequently, no behavioural pathways could be identified between company-level reciprocity and high sickness absence (2→1→1), and company-level health-related behaviours did not mediate the relation between company-level reciprocity and individual high sickness absence (2→2→1) (Table 2).

## Discussion

The present study demonstrated a relationship between workplace reciprocity and high sickness absence so that higher levels of supervisor reciprocity related to lower future sickness absence. Even though objective follow-up data improve causal inference, without a clear understanding of the underlying mechanisms, it remains a black-box operation. To address this, three types of mediation were tested for multiple health behaviours (i.e. smoking, alcohol consumption, and physical activity) in the relation between workplace reciprocity and future high sickness absence. Overall, workplace reciprocity related to sickness absence independently from health behaviours. The finding is in line with Oksanen et al. [23] who found that controlling for health behaviours had no effect on the association between workplace social capital and self-rated health, but contradicts Väänänen et al. [24] who found the opposite. However, both studies did not explicitly test for mediational pathways. We found only little evidence for an indirect behavioural pathway. The single-level path model (1→1→1) demonstrated that horizontal emotional reciprocity (colleague interest) indirectly related to sickness absence via individual smoking behaviour so that colleague interest positively related to smoking and smoking, in turn, positively related to sickness absence. Colleague interest also positively related to physical activity and physical activity, in turn, negatively related to sickness absence. However, the specific latter indirect effect was not significant. No mediational pathway was found for alcohol consumption which is in contrast to the only pathway study on hypertension in workplaces [25]. Our results complement similar studies on neighbourhood social capital which found limited or no evidence for smoking and alcohol consumption risk behaviour pathways, [29–31,52] and partly support a physical activity behavioural pathway [28,31]. Conventional social capital research tends to emphasize the positive role of social capital as a buffer for stress by enhancing the individual’s coping abilities [53], promoting social control over deviant behaviours and reinforce healthy norms [32], and increasing motivation for self-care through the positive psychological states related to social network integration [53]. Research on the negative consequences of social capital had been a relatively unexamined area of investigation, particularly compared to studies on its beneficial effects for health [54]. To date, the sparse empirical evidence from prospective studies either confirmed the beneficial influence of workplace social capital on smoking [21] or reported no relation [24]. From a social support perspective, reciprocity can be either facilitator or barrier for smoking depending upon the smoking status of the supportive others [55]. Portes [5] in a highly influential paper emphasized this so-called dark sides of social capital. Although it may be a plausible explanation in a school context where horizontal ties between pupils have shown to increase smoking [56], it is much less likely the case in an adult work environment where smoking patterns are already established in earlier years. Our tentative interpretation of the present findings is that the path between colleague interest and smoking suffers from reversed causation and that smoking status is the common cause of both reciprocity and future sickness absence: smoking colleagues have simply developed stronger ties as a result of common smoke breaks at work.

Furthermore, the multilevel path models (2→1→1 & 2→2→1) showed that individual sickness absence was mainly driven by individual factors. Differences in sickness absence between companies were not attributable to either compositional or contextual differences in reciprocity [1]. The lack of association between company-level reciprocity and sickness absence may be due to the referent area (i.e. company). Since the average cluster size varied between 103 and 2203 employees (mean = 677; SD = 574) it could have been too broad to capture the degree of reciprocity exchanges occurring within the workplace. In contrast to generalized trust, reciprocity implies a close two-way interaction, with the expectation that the favor would be returned when needed. Indeed, it may be less likely to expect something in return from coworkers belonging to work-units outsides one’s own. Informal work groups or at least smaller hierarchical units within a workplace might provide a more accurate proxy for ecological-level social capital, but no such information was available in the present study. However, given this imprecise measurement of contextual reciprocity, the present results are likely to be an underestimation of the true contextual relations of workplace reciprocity. Furthermore, it is also possible that company-level reciprocity affects individual sickness absence only if it also influences the individual’s own perception of reciprocity. Future studies should investigate this via cross-level interaction effects.

### Strengths & limitations

The systematic assessment of several (multilevel) multiple mediation models was a major strength of the present study. Our path analysis approach has advantages over the basic mediational analysis framework which involves a simple three variable system [57]. A path model additionally takes interrelations between model parameters into account which leads to more accurate results. Following social cohesion theory [32], a proper examination of social capital as a collective influence on health requires a multilevel approach. The present study addressed almost all substantial recommendations from Choi and colleagues [7] and also extended the Krull and MacKinnon [35] mediation typology in two ways. First, in imitation of Uchino et al. [58] we conceptualized a multiple mediation model consisting of several health behaviour mediators (i.e. smoking, alcohol, and physical activity). And second, we included identical individual- and company-level variables simultaneously which enables to distinguish between compositional and contextual explanations of company effects [1]. Identifying such relationships in a different study context than the Finnish Public Sector Study [21–27] by using an objective outcome instead of self-reported data contributed to the development of workplace preventive and intervention strategies.

However, several limitations of this study are noteworthy. First, although sickness absence was measured during a follow-up period of 12 months, reversed causality is still possible. Second, no objective baseline sickness absence information was available. Third, sickness absence duration, which contains no information about the frequency of absence, was used. However, we performed some additional analysis for sickness absence frequency (≥ 3 episodes). The results only differed from the reported sickness absence duration (≥ 10 days) findings such that high levels of company-level colleague interest decreased sickness absence frequency (OR = 0.149, 95% CI = 0.049, 0.450). Fourth, the study assessed health behaviours with self-reports, which can cause recall, reporting and response bias. Non-response and misclassification are likely to influence different health behaviours to differing degrees. For example, self-reported current smoking is probably more accurate than self-reported alcohol use [59]. Last, we did not assess social capital outside the workplace setting. It is highly plausible that social capital outside work affects social capital in the workplace and vice versa [13]. Future studies should examine the relative importance of different sources of social capital for health.

This study adds to previous research by showing that workplace reciprocity relates to future sickness absence, however mainly independent from health behaviours. Our findings therefore suggest that both these factors may be relevant in terms of employee health, but arguing that health behaviours are the underlying explanatory mechanism between workplace reciprocity and health seems to be a bridge too far.
