# Correction: Health Behaviours As a Mechanism in the Prospective Relation between Workplace Reciprocity and Absenteeism: A Bridge too Far ?

**DOI:** 10.1371/journal.pone.0144396

**Published:** 2015-12-01

**Authors:** Bart De Clercq, Els Clays, Heidi Janssens, Dirk De Bacquer, Annalisa Casini, France Kittel, Lutgart Braeckman

The affiliation for the sixth author is incorrect. Dr. France Kittel is not affiliated with #2 but with #3 School of Public Health, Université libre de Bruxelles, Brussels, Belgium.
